# Abemaciclib-Associated Skin, Hair, and Nail Toxicities: A Case Report

**DOI:** 10.7759/cureus.57677

**Published:** 2024-04-05

**Authors:** Hanadi Alsatti, Basel AlMalki, Yara Alghamdi

**Affiliations:** 1 Dermatology, King Abdulaziz Medical City (KAMC), Jeddah, SAU; 2 Dermatology, King Fahad General Hospital, Jeddah, SAU; 3 Dermatology, King Abdullah Medical Complex, Jeddah, SAU

**Keywords:** ribociclib, palbociclib, abemaciclib, cdk4/6i, cyclin-dependent kinase 4/6 inhibitors

## Abstract

Abemaciclib is a cyclin-dependent kinase 4/6 inhibitor that is utilized to manage hormone-sensitive human epidermal growth factor receptor-2 positive metastatic breast cancer. Palbociclib and ribociclib are types of other orally administered CDK4/6i that share similar safety and tolerability compared with Abemaciclib. Reported side effects include reversible neutropenia of a lower grade, gastrointestinal toxicity, and anemia. CDK4/6i could induce dermatological side effects. Although less frequent, cutaneous adverse effects of CDK4/6i account for 25% of medication discontinuation. Frequent cutaneous adverse events are rash and pruritus; nonetheless, hair loss, nail changes, vitiligo, and photosensitivity reactions, were also reported to a lesser extent. Herein, we report a case of hair, nail, and pigmentary disorder that occurred 10 months after initiating abemaciclib treatment.

## Introduction

Cyclin-dependent kinase 4/6 inhibitors (CDK4/6i) are routinely used in treating hormone-sensitive human epidermal growth factor receptor-2 (HER2) positive metastatic breast cancer [1]. These inhibitors are crucial in regulating cell cycle transitions and cell lineage commitments [2]. Cyclin-dependent kinases (CDKs) include CDK1, CDK2, CDK3, CDK4, and CDK6. These CDKs are key essential steps in cell division and commitment to cell lineage [2]. In human and animal studies, the overall control of cell cycle entry is dependent on CDK4 and CDK6 [2]. Retinoblastoma protein (Rb) is a key negative regulator of cellular division. On a molecular level, RB is phosphorylated by either CDK4 or CDK6, which leads to cell cycle progression [2]. In terms of therapeutics, inhibition of CDK4 and CDK6 would be of interest, as it would disrupt the growth of the cellular axis [2]. The development of selective inhibitors of both CDK4 and CDK6 has changed the understanding of these molecules. In numerous trials, estrogen-sensitive tumor cells have been suppressed following the administration of CDK4/6i [1-3].

Palbociclib, ribociclib, and abemaciclib are orally administered types of CDK4/6i with similar effectiveness, though their safety and tolerability vary. Common side effects include gastrointestinal toxicity, reversible neutropenia of a lower grade, and anemia [1]. Abemaciclib is often linked with poorer tolerability and a higher rate of treatment discontinuation due to gastrointestinal toxicity, primarily diarrhea and fatigue [1,2]. Dermatological reactions to CDK4/6i, reported both during clinical trials and post-market, although less frequent, accounted for 15% of all adverse effects, with 25% of these requiring medication termination [3]. In this report, we highlight a case of dermatological side effects, specifically hair loss, hyperpigmentation, and nail changes, which occurred 10 months after initiating abemaciclib treatment.

## Case presentation

A 39-year-old female patient who suffers from dyslipidemia and stress-induced cardiomyopathy was diagnosed with bilateral breast cancer. The cancer, which showed a BRCA-1 gene mutation and positive ER and PR receptors but a negative HER-2 receptor, prompted several treatments. These treatments included chemotherapy, a bilateral skin-sparing modified radical mastectomy, salpingo-oophorectomy, radiation therapy, and immunotherapy with abemaciclib and letrozole.

**Figure 1:**
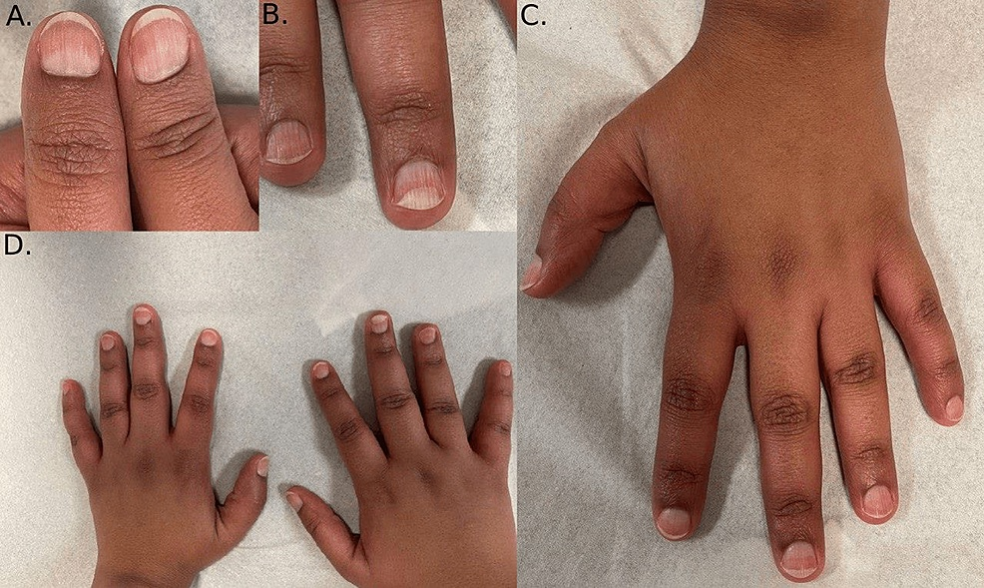
(A and B) Both middle fingernails displayed onycholysis; (C and D) All nails demonstrated roughness and longitudinal ridges (trachonychia)

**Figure 2:**
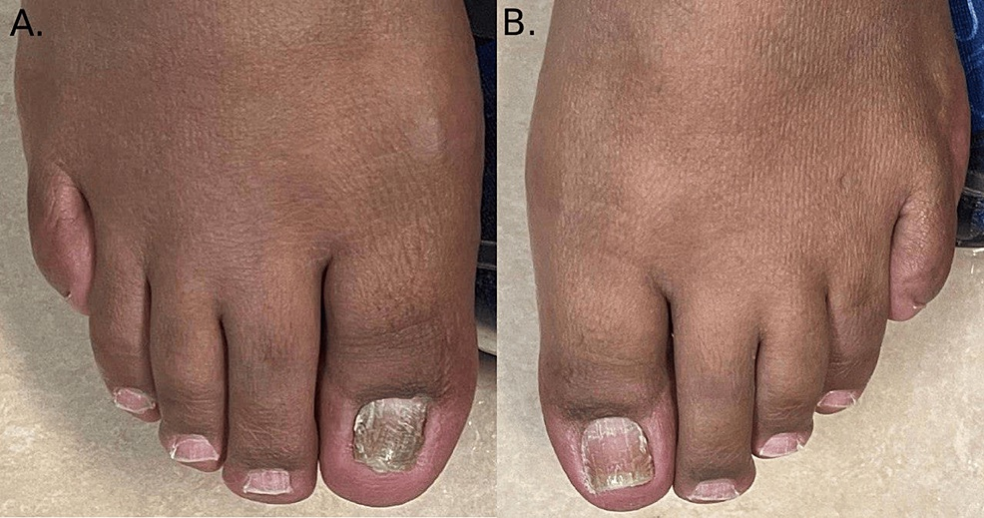
Both big toenails are thick, rough, and hyperkeratotic with proximal separation in the right big toenail

Fifteen months before visiting our clinic, she started immunotherapy. Alongside that, the patient was also managing her cardiomyopathy with Entresto. Within 10 months of starting the immunotherapy, she developed onycholysis of her nails. Her nails were noticeably rough with longitudinal ridges (Figure 1). More specifically, her middle fingers had onycholysis (Figure 1), while her big toenails were thick and coarse with subungual hyperkeratosis; the right toenail has started to separate (Figure 2). However, tests for fungal infection of the nails came back negative.

**Figure 3:**
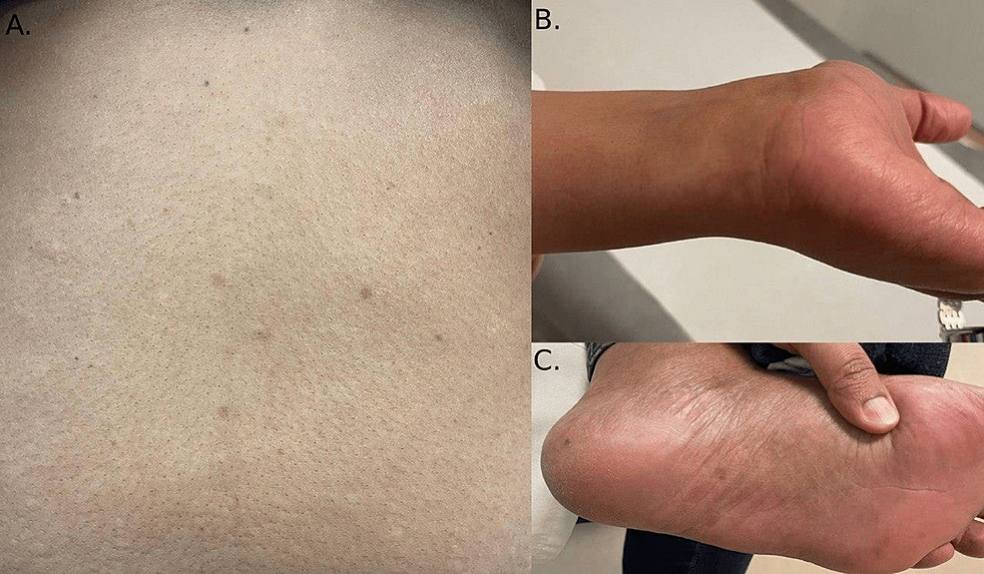
(A) Scattered hyperpigmentation macules over the trunk; (B and C) There is hyperpigmentation in the palms and soles

Further physical observations included hyperpigmentation in her palms and soles (Figure 3), as well as increased hair shedding that started 6 months before our inspection. Lower hair density was noticeable around the temporal regions of her scalp. Nevertheless, her ferritin, thyroid function, and vitamin D levels were all normal.

These symptoms suggested drug-induced nail changes and pigmentation. Like her current situation, she had experienced similar nail changes (trachonychia) during her chemotherapy, which were managed with topical amorolfine solution. Currently, her treatment involves once-a-week amorolfine and emollient applications for symptom relief.

The nail and hair manifestations have developed progressively within 6-10 months after initiating abemaciclib. Necessary laboratory and other workups were done; however, abemaciclib-induced hair, nail, and skin toxicities were the culprit. Although the patient was on amorolfine solution (once weekly) and topical emollient for her nails, there was no noticeable improvement. Despite these bothersome symptoms, they did not affect quality of life.

Although abemaciclib likely caused the hair, nail, and skin conditions, the patient managed to tolerate them. On these clinical grounds, discontinuation of the treatment was not indicated, as these side effects should reverse themselves upon stopping the drug.

## Discussion

On a molecular level, a key stage in cellular division is the transition from the G1 to the S phase, which occurs through the inactivation of the retinoblastoma protein, a regulatory protein that prevents early cell division. Cyclin-dependent kinases (CDK) 4 and CDK 6 are serine/threonine kinases that phosphorylate this protein, enabling cellular division to proceed. Thus, therapeutically, the combined inhibition of these kinases could exhibit an anticancer effect [1]. The most frequently reported cutaneous adverse event (AE) following CDK4/6 inhibitors is alopecia, followed by rash and pruritus [3]. Grade 1 alopecia has been reported with palbociclib and ribociclib, affecting 33% of patients as compared to 16% in other treatment groups [2].

A large number of skin-related AEs were reported following the use of abemaciclib and palbociclib [3]. Common skin reactions to abemaciclib included hair loss (20.7%) and rashes (12.9%) [2,3]. The onset of rashes often occurred within the first 28 days of starting the CDK4/6i treatment while changes to hair and nails typically appeared between 67 and 112 days [3].

Ribociclib was associated with pruritus, various types of cutaneous exanthems, vitiligo, and photosensitivity reactions [3,4]. Pruritus and maculopapular rashes were mainly reported among patients receiving ribociclib and palbociclib, with only a few reports in abemaciclib recipients [3]. Bullous conditions, such as bullous dermatitis, were reported with ribociclib and bullous pemphigoid with palbociclib while erythema multiforme was more commonly associated with abemaciclib [3].

Post-marketing AEs have shown that erythema multiforme is reported more frequently with abemaciclib and palbociclib [5]. Palbociclib has been linked to dermatological and epidermal conditions, including xerosis, nail bed disorders, such as onycholysis, and hair disorders like trichorrhexis [3].

Raschi et al. reported the adverse effects of CDK4/6i agents [3]. They found that nail and nail bed disorders occurred most frequently with palbociclib and then ribociclib. Abemaciclib was only reported to cause onycholysis in a few cases, and no nail ridging or nail growth abnormalities were reported with it. Notably, our patient developed nail manifestations with abemaciclib, and changing to another drug in the same class did not alleviate these symptoms.

Pilar disorders were predominantly linked to palbociclib [3]. Conversely, abemaciclib had no reported association with abnormal hair growth or trichorrhexis. Reports of subacute cutaneous lupus erythematosus emerged in relation to ribociclib and abemaciclib [6-9].

Salusti-Simpson et al. reported abemaciclib-induced hyperpigmentation in a 64-year-old female with a medical history of invasive right breast adenocarcinoma [10]. The patient was on a chronic regimen of anastrozole and abemaciclib, and the diffuse hyperpigmentation might be attributed to abemaciclib [10]. Cutaneous manifestations elicited by CDK4/6 inhibitors are likely to improve with drug discontinuation [1].

Less frequently reported serious AEs associated with CDK4/6 inhibitors include Stevens-Johnson syndrome, toxic epidermal necrolysis, Henoch-Schonlein purpura, radiation recall dermatitis, histiocytoid Sweet syndrome, erythema dyschromicum perstans, vitiligo-like lesions, and cutaneous leukocytoclastic vasculitis [11,12].

Discontinuing treatment significantly impacts the efficacy of anticancer drugs. A study found that 25% of discontinuations of CDK4/6i were due to skin reactions [3]. The most commonly reported side effects are often non-specific and do not typically present serious adverse effects. However, their chronic nature, visibility in areas impacting one's appearance, and bothersome symptoms like pain or itchiness require immediate attention and management. These side effects can potentially harm patients' treatment compliance and quality of life. In our patient, we opted not to discontinue anticancer treatment as cutaneous manifestations were mild and there was no indication for cessation. We elected not to dechallange and rechallange the patient, as we did not want to risk cancer relapse.

## Conclusions

Post-market surveillance of adverse effects is essential in establishing the safety profile of a drug and in raising awareness about possible side effects. As far as we are aware, there are a few reported cases demonstrating hair, nail, and skin toxicities after using abemaciclib. Skin toxicities connected with CDK4/6i are currently under-reported, and we encourage similar future reports to ensure the safe use of such new drugs.
